# A single‐cell survey of the human glomerulonephritis

**DOI:** 10.1111/jcmm.16407

**Published:** 2021-03-22

**Authors:** Zhejun Chen, Ting Zhang, Kaiqiong Mao, Xinghua Shao, Yao Xu, Minyan Zhu, Hang Zhou, Qin Wang, Zhenyuan Li, YuanYuan Xie, Xiaodong Yuan, Liang Ying, Ming Zhang, Jiajia Hu, Shan Mou

**Affiliations:** ^1^ Department of Nephrology Molecular Cell Laboratory for Kidney Disease Ren Ji Hospital School of Medicine Shanghai Jiao Tong University Shanghai China; ^2^ Shanghai Institute of Immunology Department of Microbiology and Immunology Shanghai Jiao Tong University School of Medicine (SJTU‐SM) Shanghai China; ^3^ Transplantation Center of Ren Ji Hospital School of Medicine Shanghai Jiao Tong University Shanghai China; ^4^ Department of Nuclear Medicine Ruijin Hospital Shanghai Jiao Tong University School of Medicine Shanghai China

**Keywords:** kidney disease, nephritis, single‐cell RNA‐seq, transcriptomics

## Abstract

Glomerulonephritis is the one of the major causes of the end‐stage kidney disease, whereas the pathological process of glomerulonephritis is still not completely understood. Single‐cell RNA sequencing (scRNA‐seq) emerges to be a powerful tool to evaluate the full heterogeneity of kidney diseases. To reveal cellular gene expression profiles of glomerulonephritis, we performed scRNA‐seq of 2 human kidney transplantation donor samples, 4 human glomerulonephritis samples, 1 human malignant hypertension (MH) sample and 1 human chronic interstitial nephritis (CIN) sample, all tissues were taken from the biopsy. After filtering the cells with < 200 genes and > 10% mitochondria (MT) genes, the resulting 14 932 cells can be divided into 20 cell clusters, consistently with the previous report, in disease samples dramatic immune cells infiltration was found, among which a proximal tubule (PT) subset characterized by wnt‐β catenin activation and a natural killer T (NKT) subset high expressing LTB were found. Furthermore, in the cluster of the podocyte, three glomerulonephritis related genes named FXYD5, CD74 and B2M were found. Compared with the mesangial of donor, the gene CLIC1 and RPS26 were up‐regulated in mesangial of IgA nephropathy(IgAN), whereas the gene JUNB was up‐regulated in podocyte of IgAN in comparison with that of donor. Meanwhile, some membranous nephropathy (MN) high expressed genes such as HLA‐DRB5, HLA‐DQA2, IFNG, CCL2 and NR4A2, which involve in highest enrichment pathway, display the cellular‐specific expression style, whereas monocyte marker of lupus nephritis (LN) named TNFSF13B was also found and interferon alpha/beta signalling pathway was enriched in B and NKT of LN comparing with donor. By scRNA‐seq, we first defined the podocyte markers of glomerulonephritis and specific markers in IgA, MN and LN were found at cellular level. Furthermore, the critical role of interferon alpha/beta signalling pathway was enriched in B and NKT of LN was declared.

**Abbreviations**
APRIL: a proliferation‐inducing ligand;
B2M: ß2‐microglobulin;
CD: collecting duct;
CIN: chronic interstitial nephritis;
CKD: chronic kidney disease;
CLIC1: chloride intracellular channel 1
DCT: distal tubule;
EC: endothelial cell;
GWAS: Genome‐wide association studies;
IgAN: IgA nephropathy;
LN: lupus nephritis;
LOH: loop of henle;
MH: malignant hypertension;
MIF: Macrophage migration inhibitory factor;
MN: membranous nephropathy;
MT: mitochondria;
NKT: natural killer T;
PLA2R: Podocyte phospholipase A2 receptor;
PT: proximal tubule;
scRNA‐seq: Single‐cell RNA sequencing;
SLE: systemic lupus erythematosus;

## 1 INTRODUCTION

The kidney is a highly complex tissue with a broad range of specialized cell types organized into functionally distinct compartments. Bulk RNA‐seq cannot detect heterogeneity of renal cells; thus, specific renal cells functioning abnormally may be covered by the majority of normal cell types.^1^

Glomerulonephritis is one of the major reasons leading to renal failure. The clinical manifestation of glomerulonephritis is a heterogeneous group of diseases, which were diagnosed by biopsy. However, specific pathogenesis underlying histological findings seems not to be totally understood.^2^

As one of the most common renal diseases, IgA nephropathy (IgAN) is characterized by the deposition of IgA immune complexes in glomerulus. The mesangial IgA is exclusive of the IgA1 subclass, which is mostly in a dimeric form that is composed of two IgA1 monomers and J chain.^3^ Genome‐wide association studies (GWAS) have found IgAN contributing genes including *HLA‐DRB1*, *‐DQA1* and *DQB1*, which mediate the MHC class II response. Besides, *TAP1*, *TAP2*, *PSM8* and *PSM9* involved in antigen generation are also IgAN associated genes.^4^ CFH and the related genes contain a protective role via inhibiting the C3 and C5 convertases, which promote the complement cascade. Chinese GWAS found a new loci centred on gene *TNFSF13* encoding a proliferation‐inducing ligand (APRIL) that influences IgA‐producing cells.^5^ However, whether these IgAN associated genes expressed abnormal comparing with healthy kidney at cellular level is still unknown.

Membranous nephropathy (MN), which is the second or third leading cause of end‐stage renal disease in patients with primary glomerulonephritis,^6^ is a major cause of nephrotic syndrome of non‐diabetic origin in adults. The disease is characterized by the formation of immune deposits on the outer layer of the glomerular basement membrane resulting in complement activation. Podocyte phospholipase A2 receptor (PLA2R) was found as an antigenic target in autoimmune adult MN. Measurement of anti‐PLA2R antibodies in serum and detection of PLA2R antigen in glomerular deposits can be a very good indicator for the diagnosis and treatment of MN.^6^ However, in addition to primary MN (caused by autoantibody), there have other forms of MN in which anti‐PLA2R antibodies cannot be detected.^7^ ScRNA‐seq may have a role in evaluating the mechanisms underlying the common clinical manifestations and common complement activation.

Lupus nephritis(LN) is a common and serious complication of systemic lupus erythematosus (SLE). Thus far, renal biopsy remains to be the standard for the severity of LN as current laboratory markers for LN lack sensitivity and specificity for renal disease activity and damage in LN.^8^ As such, novel biomarkers should be able to act as surrogate indicators instead of an invasive method. However, these functionally disease‐related genes originating from specific cell type remain unknown. The focus of this study is to find whether the diseases‐associated genes originate from specific cell type.

## 2 METHODS

### 2.1 Clinical sample

We adopt two transplantation donor biopsy samples from transplantation centre as normal controls, whereas other samples acquired from the nephrology department were used as a group of kidney diseases whose tissues were taken from biopsy to confirm their cause of proteinuria or rise in the serum creatinine. As the pathological results show, one donor sample and two patients were confirmed as IgA nephropathy with other four patients diagnosed as lupus nephritis, membranous nephropathy, malignant hypertension and chronic interstitial nephropathy, respectively, whereas another donor sample was relatively normal besides reperfusion injury. The patient described (Table 1) in this study consented under the ethics committee review of Renji Hospital affiliated to Shanghai Jiao Tong University School of Medicine.

**TABLE 1:** Clinical Demographics of biopsy samples

Sample ID	age	sex	Serum creatinine(uM)	eGFR‐EPI Cr	24 h proteinuria	complications
Donor1(IgA)	46	male	50	NA	NA	Glu 19.77 mmol/L RBC by urine sediment:211/ul
Donor2	NA	male	88	NA	NA	NA
IgAN1	61	male	107.9	NA	1.19g	RBC:17/HP
LN	27	male	145	56	NA	NA
MN	60	male	62	103	16g	NA
CIN	62	male	203	29	NA	renal arteriolar sclerosis HbA1c:10.0
IgAN2	38	female	117	72	2.075g	RBC:22.1/HP HBV‐DNA:1.14E + 05
MH	38	male	184	39	NA	NA

Abbreviation

NA: Not Available.

### 2.2 Tissue processing and single‐cell dissociation

The renal biopsy was preserved in cold PBS(1X), then minced into small pieces with a razor blade and incubated at 37°C in freshly prepared dissociation buffer containing enzymes from Multi Tissue Dissociation Kit (Miltenyi Biotec) with rotation speed of 200rpm. Dissociated cells were harvested every 10 minutes by filtering the cell suspension through a 70‐µm cell strainer (FALCON) into 10% FBS buffer on ice. The residual biopsy tissue was dissociated once again with 1 ml dissociation buffer for 10 minutes and passed through the cell strainer into the same FBS buffer from the first collection. This dissociation procedure was repeated three times until most of the tissue had been separated into single cells (total dissociation time was 30 minutes). Finally, cells were collected by centrifugation at 1500 rpm for 5 minutes, resuspended in PBS(1X) containing 5% FBS and strained through a 40‐µm cell strainer (FALCON) to further remove cell clumps and large fragments. Cell viability was approximately 85% for the biopsy used in this study as assessed by Trypan Blue staining.

### 2.3 Single‐cell RNA sequencing, library construction and bioinformatic analysis

According to manufacturer's(10X Genomics) protocol, single‐cell suspensions were used to generate libraries with the Chromium Single‐Cell Gene Expression system using 3′ Library& Gel Bead Kit v2 (10X Genomics) and paired‐end sequencing was performed on a HiSeq. The raw sequencing reads from 10x Genomics 3‐prime sequencing were processed with Cell Ranger to generate gene‐cell count matrices. This matrix was filtered retaining cells with more than 200 genes and less than 10% mitochondria transcripts. According to the instructions of SATIJALAB (https://satijalab.org/seurat/), data sets from kidney samples were integrated with Seurat using canonical correlation analysis (CCA); then, the calculation of 2000 high‐variance genes, data normalization with log (Counts per 10,000 + 1), scaling, dimensional reduction, graph‐based clustering and the identification(Wilcoxon test by default) of cluster markers was completed. UMAP was used for 2‐dimensional visualization.^9^, ^10^

### 2.4 Gene enrichment analysis

The differential genes (average FC > 1.0 or 1.5, p value < 0.05) were extracted to finish gene enrichment analysis, whereas gene enrichment analysis was completed through metascape (http://metascape.org/gp/index.html#/main/step1) ^11^ by input the gene list.

## 3 RESULTS

### 3.1 Overview of cell heterogeneity in integrated 8 samples and 20 cell clusters were identified

**FIGURE 1:**
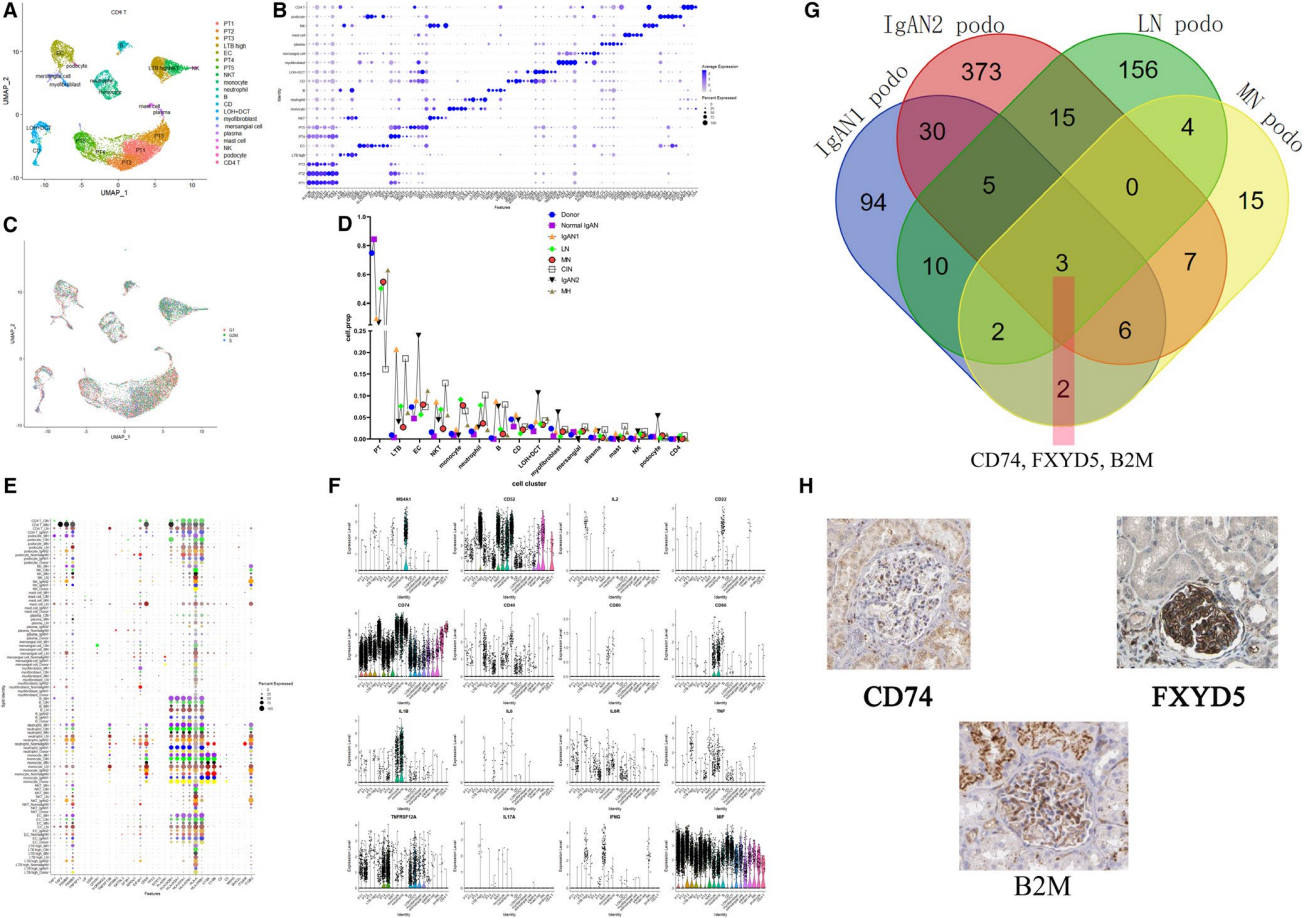
Overview of cell heterogeneity in integrated 8 samples and 20 cell clusters were identified. Samples of two kidney transplantation donors, two IgAN, one LN, one MN, one MH and one CIN were merged. One of the donors was confirmed as IgA nephropathy by immunohistochemistry. (A) And 20 cell clusters including 5 types of proximal tubules (PT), loop of henle (LOH)+distal tubule (DCT), collecting duct (CD), endothelial cell(EC), monocyte, myofibroblast, mesangial, B cell, podocyte, NKT, NK, LTB high, CD4, mast cell and plasma cell were found. (B) The marker genes of each cell clusters. (C) The umap shows the cell cycle status of each cell clusters. (D) The figure shows cell proportion of different kidney samples across 20 cell clusters. (E) GWAS genes and (F) target genes of promising biologics in the specific cell clusters. Dot plot shows cellular‐specific expression of GWAS genes,^14^, ^44^ and vlnplot shows the target genes of current and evolving biologics expressed in specific cell types.^45^ (G) The higher expressed gene in podocyte of MN, LN, IgAN comparing with that in donor was merged in Venn diagram, (H) IHC staining of common high expressed three gene *CD74* (https://www.proteinatlas.org/ENSG00000019582-CD74/tissue/kidney#img), *FXYD5* (https://www.proteinatlas.org/ENSG00000089327-FXYD5/tissue/kidney#img) and *B2M* (https://www.proteinatlas.org/ENSG00000166710-B2M/tissue/kidney#img), which is from the Human Protein Atlas (https://www.proteinatlas.org/), was shown

After filtering the cells with < 200 genes and > 10% MT genes, the resulting 14 932 cells(Figure 1A) can be divided into 20 cell clusters including 5 types of proximal tubules (PT), a loop of henle (LOH)+distal tubule (DCT), collecting duct (CD), endothelial cell(EC), monocyte, myofibroblast, B cell, podocyte, mesangial cell, neutrophil, LTB high, NK, CD4 T, NKT, mast cell and plasma cell and labelled each cluster by expression of known marker genes(Figure 1B). In order to evaluate the cell cycle status of each cell cluster, B and NK cell seems to be at the status of cell division comparing with other cell types (Figure 1C). By calculating the cell proportion across 8 samples, decreased PT cell clusters and increased immune cell clusters in diseased samples comparing with two donors were found (Figure 1D). Moreover, an inflammatory PT subtype named PT5 was also found in our data set. The top genes (defined as high fold change) of PT5 were displayed (Table 2). The wnt‐β catenin target genes (*MMP7* AND *MYC*) were also activated in PT5, as shown by red colour (Table 2).

**TABLE 2:** The top genes, defined as high fold change, of PT5

gene	avg_logFC	pct.1	pct.2	p_val_adj
PIGR	1.456872162	0.77	0.164	0
SOX4	1.314714694	0.746	0.144	0
CLU	1.258028352	0.848	0.326	6.79E‐209
CD24	1.142787645	0.87	0.314	0
NEAT1	1.128487256	0.906	0.385	6.74E‐230
MMP7	1.101394271	0.571	0.072	0
KRT18	1.088693148	0.862	0.263	0
LTF	1.023991598	0.305	0.084	3.51E‐100
CDH6	1.019785935	0.636	0.068	0
VCAM1	1.014635852	0.708	0.104	0
TSPAN1	0.976341777	0.843	0.246	0
CCL2	0.970346357	0.526	0.117	3.49E‐242
ITGB8	0.965309883	0.67	0.071	0
AKAP12	0.965302964	0.62	0.076	0
TNFSF10	0.950990028	0.808	0.219	0
ACSM2A	0.902459008	0.776	0.157	0
C19orf33	0.894327065	0.514	0.063	0
AQP1	0.88775269	0.765	0.206	0
TPM1	0.848070458	0.846	0.296	5.89E‐272
NTN4	0.847401666	0.611	0.08	0
MT1E	0.820899321	0.851	0.521	4.13E‐72
RHOB	0.818744508	0.772	0.273	6.71E‐225
TNFRSF12A	0.817793412	0.692	0.129	0
KLF6	0.806595439	0.762	0.284	2.29E‐192
REG1A	0.789675292	0.277	0.08	2.09E‐79
KRT8	0.781275707	0.85	0.288	2.90E‐253
LINC01320	0.76968022	0.685	0.126	0
CYR61	0.763999118	0.563	0.051	0
UGT2A3	0.752457212	0.683	0.103	0
CLDN3	0.751093152	0.605	0.092	0
ELF3	0.748905106	0.657	0.12	0
SOX9	0.722433434	0.534	0.071	0
ATF3	0.711958526	0.479	0.099	8.45E‐235
GSTP1	0.711458628	0.959	0.604	5.80E‐145
SPINT2	0.705663959	0.796	0.255	8.79E‐244
VIM	0.697470937	0.775	0.311	8.33E‐166
CXCL1	0.694321279	0.304	0.023	0
VMP1	0.689420973	0.684	0.162	1.13E‐292
C12orf75	0.684124779	0.881	0.394	4.67E‐188
TMEM176A	0.681654207	0.897	0.39	8.89E‐193
TMEM176B	0.678384266	0.877	0.367	4.71E‐185
BICC1	0.670233608	0.626	0.106	0
EGR1	0.661963807	0.58	0.143	1.92E‐226
LEPROT	0.661427612	0.747	0.232	9.00E‐240
ACSM2B	0.652503595	0.742	0.201	2.33E‐260
CPM	0.646770512	0.577	0.088	0
TMEM27	0.642617266	0.775	0.237	1.05E‐223
THBS1	0.642265793	0.498	0.059	0
UGCG	0.641257087	0.606	0.108	0
SLC22A2	0.63698695	0.62	0.09	0
LAPTM4A	0.633164691	0.913	0.473	1.46E‐152
SERPINE2	0.629510186	0.454	0.08	1.55E‐257
MGST1	0.617604635	0.87	0.354	1.17E‐171
ANPEP	0.61613211	0.734	0.209	3.13E‐245
NAT8	0.615722142	0.881	0.442	3.82E‐105
PPFIBP1	0.615233362	0.566	0.09	0
ZFP36L1	0.610788871	0.763	0.315	2.39E‐146
SLC3A1	0.610119309	0.656	0.159	8.68E‐267
MT1 M	0.609877738	0.303	0.1	1.12E‐68
MT2A	0.609870735	0.913	0.682	2.01E‐39
CLDN4	0.603896748	0.55	0.086	0
HSP90B1	0.602853252	0.777	0.315	1.73E‐150
MDK	0.602338585	0.499	0.085	0
SYCE1L	0.591290595	0.534	0.071	0
ITM2B	0.581524653	0.983	0.759	1.05E‐130
AMN	0.578692633	0.673	0.141	0
THY1	0.576573085	0.572	0.141	4.76E‐218
OCIAD2	0.574136554	0.935	0.481	1.26E‐151
C1orf186	0.572945046	0.552	0.06	0
STAT1	0.572887165	0.515	0.142	8.79E‐168
MYC	0.570639809	0.417	0.034	0
MT‐CO1	0.569708494	0.999	0.979	1.34E‐178
CXCL14	0.567994122	0.955	0.521	1.39E‐112
GDF15	0.566626122	0.475	0.097	4.36E‐229
SDC4	0.563266371	0.659	0.157	5.60E‐270
FAM134B	0.560540687	0.564	0.094	0
SOD2	0.560087487	0.847	0.382	2.87E‐142
CEBPD	0.558068229	0.657	0.203	2.68E‐189
S100A10	0.557689277	0.882	0.464	5.23E‐117
APLP2	0.554358104	0.804	0.298	3.86E‐179
ATP1B1	0.553591296	0.93	0.457	5.41E‐149
SOCS3	0.548727895	0.507	0.129	4.16E‐182
RND3	0.523182849	0.331	0.039	1.52E‐285
CREB5	0.519938732	0.459	0.041	0
EPCAM	0.518474126	0.697	0.171	2.98E‐261
HCFC1R1	0.515076783	0.78	0.288	4.46E‐177
NFKBIZ	0.508162617	0.507	0.096	1.90E‐268
CD151	0.504398462	0.735	0.226	4.91E‐215

As we know, many GWAS study have analysed some gene mutation is associated with disease occur, development. And biologics targeting these genes demonstrated good curative effect. In order to find whether the expression of these genes is cell specific. The dot plot shows that the GWAS genes focus on clusters of glomerulus and immune cells (Figure 1E). The target genes of current and evolving biologics expression profile across different cell clusters are showed (Figure 1F). Additionally, IgAN, LN and MN are three types of glomerulonephritis which affects podocyte in the end. To investigate the common genetic change at podocyte level, thus, Venn diagram was used to find the common activated genes of IgAN, LN and MN compared with donor, and then, *FXYD5*, *CD74* and *B2 M* were found (Figure 1G). To verify the single‐cell analysis, IHC staining of the three genes, which is activated in podocyte of LN, MN, IgAN1 and IgAN2, can be detected in glomerulus^12^ (Figure 1H). And from Renal Gene expression database (http://rged.wall‐eva.net/), a higher mRNA level of CD74, B2 M and FXYD5 in IgAN or LN kidney sample comparing with control living donor was found (Extended figure 3).^13^ Therefore, our scRNA‐seq data gave a comprehensive overview about the cellular heterogeneity of glomerulonephritis, which highlights the common genetic change of glomerulonephritis at podocyte level.

### 3.2 IgAN associated genes were found at the cellular level

**FIGURE 2:**
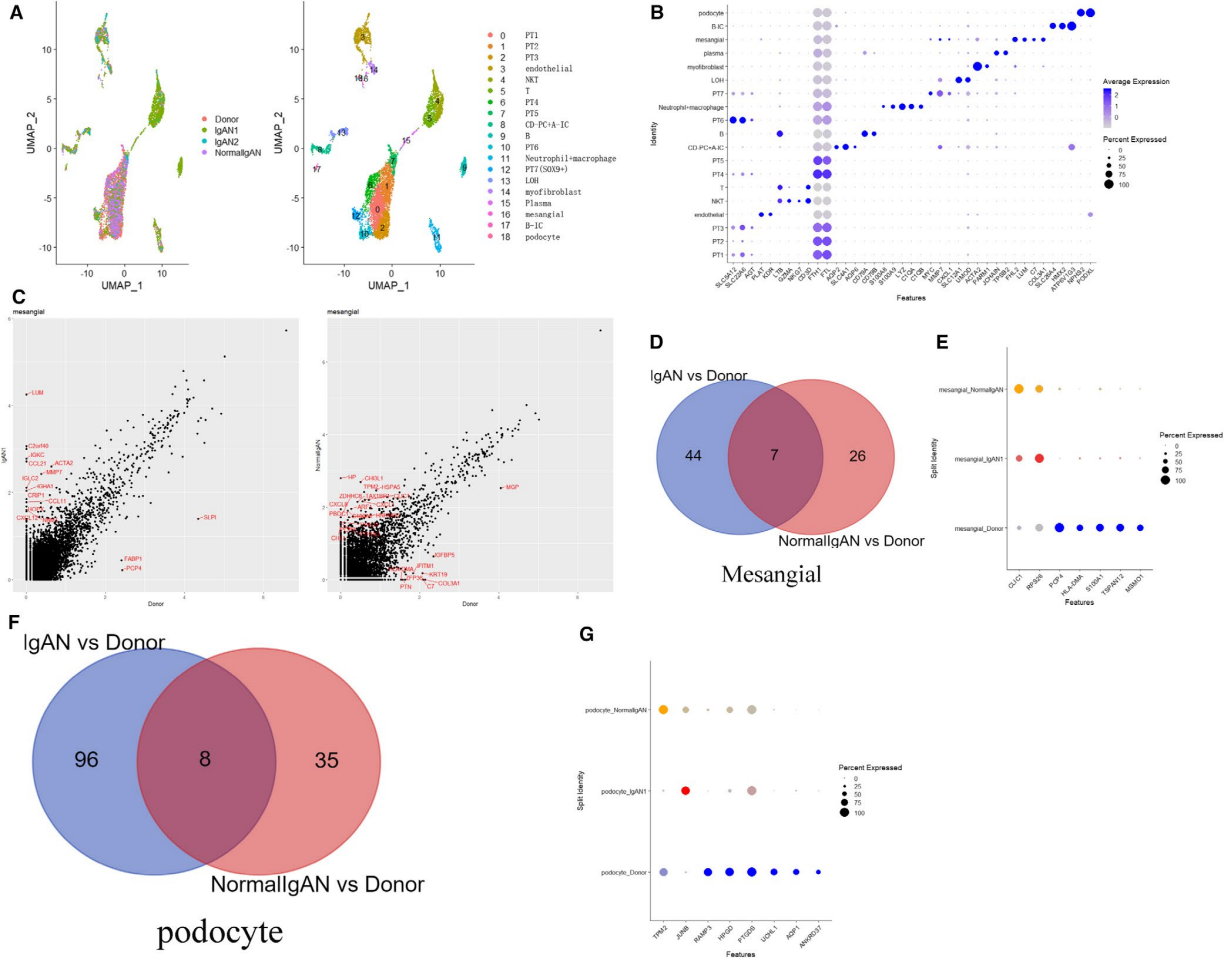
IgAN associated genes at the cellular level were shown. (A) Two IgAN, one donor confirmed as IgAN and one healthy donor, were merged. The UMAP showed the cell clusters among different samples. (B) The marker genes of each cell clusters. (C) As mesangial cells were significantly affected in IgAN, we want to see the differentially expressed genes of mesangial cell in IgAN comparing with that in donor control. The volcano plot showed differential genes (average FC > 1.5, p value < 0.05) between IgAN comparing with Donor within mesangial. (D) Differentially expressed genes (average FC > 1.0, p value < 0.05) of IgANs comparing with donor were merged in Venn diagram, and there are 7 common differentially expressed genes. (E) The expression level of the 7 genes was shown in IgAN, NormalIgAN and Donor by dot plot, and we can see the gene CLIC1 and RPS26 were up‐regulated in mesangial of IgAN compared with the mesangial of donor. As podocyte was finally affected in glomerulonephritis such as IgAN, (F) then differentially expressed genes (average FC > 1.0, p value < 0.05) within podocyte of IgANs comparing with that of donor were merged in Venn diagram, and there are 8 common differentially expressed genes. (G) The expression level of the 8 genes within podocyte was shown in IgAN, NormalIgAN and Donor by dot plot

To investigate cellular‐specific IgAN associated genes, two IgAN, one donor confirmed as IgAN and one healthy donor, were merged. The UMAP showed the cell clusters among different samples (Figure 2A). The marker genes of each cell clusters are showed by dot plot (Figure 2B). As the immune complex was deposited in mesangial cell of IgAN, the genes differentially expressed in mesangial cells of IgAN comparing with donor control were, respectively, showed by the volcano plot and Venn diagram. The volcano plot showed differential genes (average FC > 1.5, p value < 0.05) between IgAN comparing with Donor within mesangial (Figure 2C). Differentially expressed genes (average FC > 1.0, p value < 0.05) of IgANs comparing with donor were merged in the Venn diagram (Figure 2D), and there were 7 common differentially expressed genes. The expression level of the 7 genes was shown in IgAN, NormalIgAN and Donor by dot plot (Figure 2E). As podocyte was finally affected in glomerulonephritis such as IgAN, then differentially expressed genes (average FC > 1.0, p value < 0.05) within podocyte of IgANs comparing with donor were merged in Venn diagram (Figure 2F), and there were 8 common differentially expressed genes. The expression level of the 8 genes within podocyte was shown in IgAN, NormalIgAN and Donor by dot plot (Figure 2G). These data gave a clue that some novel IgAN associated genes were found at cellular level, and further study needs to be performed to figure out their function.

### 3.3 MN associated genes were found at the cellular level

**FIGURE 3:**
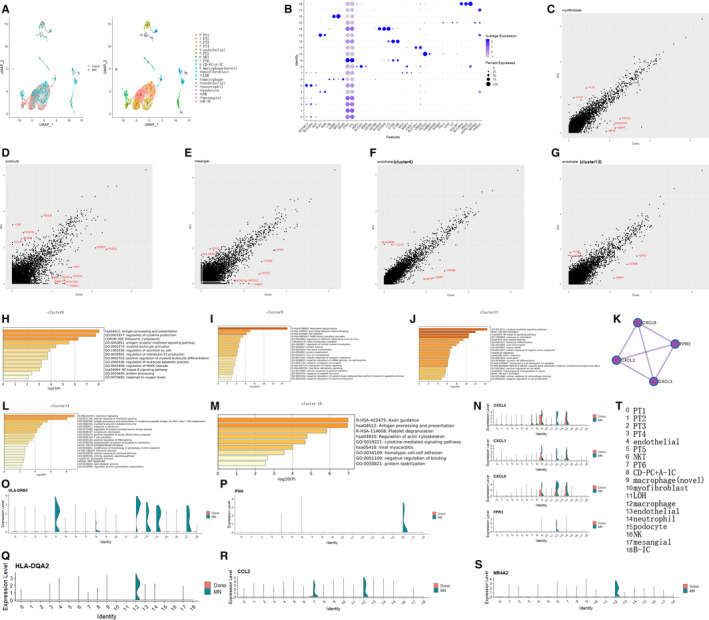
MN associated genes at cellular level were shown. (A) One MN and one healthy donor were merged. The UMAP showed the cell clusters among different samples. (B) The marker genes of each cell clusters. Volcano plot, respectively, showed differentially expressed genes between MN and donor control within myofibroblast (C), podocyte (D), mesangial (E), endothelial (cluster 4) (F) and endothelial (cluster13) (G). Gene enrichment analysis of immune cells was performed. The results, respectively, showed pathways enriched by up‐regulated genes (p value < 0.05) of MN compared with those of donor control within cluster 6(H), cluster 9(I), cluster 12(J), cluster 14(L) and cluster 16(M). (K) The interaction of chemokines and the receptor, which was up‐regulated in cluster 12 of MN compared with that of donor, was shown. (N) The differential expression level of *CXCL2*, *CXCL3*, *CXCL8* and *FPR3* between MN and donor was shown by vlnplot. Some MN high expressed genes such as *HLA‐DRB5*(O), *HLA‐DQA2*(Q), *IFNG*(P), *CCL2*(R) and *NR4A2*(S), which involve in highest enrichment pathway, were shown by vlnplot to display the cellular‐specific expression style. (T) The name of each cell cluster

To investigate cellular‐specific MN associated genes, one MN and one healthy donor were merged. The cell clusters among different samples are showed by the UMAP (Figure 3A). The marker genes of each cell cluster are showed by dot plot (Figure 3B). Volcano plot, respectively, showed differentially expressed genes within myofibroblast (Figure 3C), podocyte (Figure 3D), mesangial (Figure 3E), endothelial (cluster 4) (Figure 3F) and endothelial (cluster13) (Figure 3G) between MN and donor control. Then, gene enrichment analysis of immune cells was carried out. The results, respectively, showed up‐regulated genes (p value < 0.05) of MN compared with donor control within cluster 6 (Figure 3H), cluster 9 (Figure 3I), cluster 12 (Figure 3J), cluster 14 (Figure 3L) and cluster 16 (Figure 3M). The interaction of chemokines and the receptor, which was up‐regulated in cluster 12 of MN compared with that of the donor, was shown in figure 3K. The differential expression level of *CXCL2*, *CXCL3*, *CXCL8* and *FPR3* between MN and donor was showed by vlnplot (Figure 3N). Some previous reported MN high expressed genes such as *HLA‐DRB5* (Figure 3O), *HLA‐DQA2* (Figure 3Q), *IFNG* (Figure 3P), *CCL2* (Figure 3R) and *NR4A2*( Figure 3S), which were involved in the highest enrichment pathway, were shown by vlnplot to display the cellular‐specific expression style. These data demonstrated that *CXCL2/CXCL3/CXCL8*/*FPR3*/*CCL2*/*NR4A2* are high expressed in macrophage of MN compared with that of donor, giving a clue that more study needs to focus on these genes of macrophage among MN diseases.

### 3.4 LN‐related genes were found at the cellular level

**FIGURE 4:**
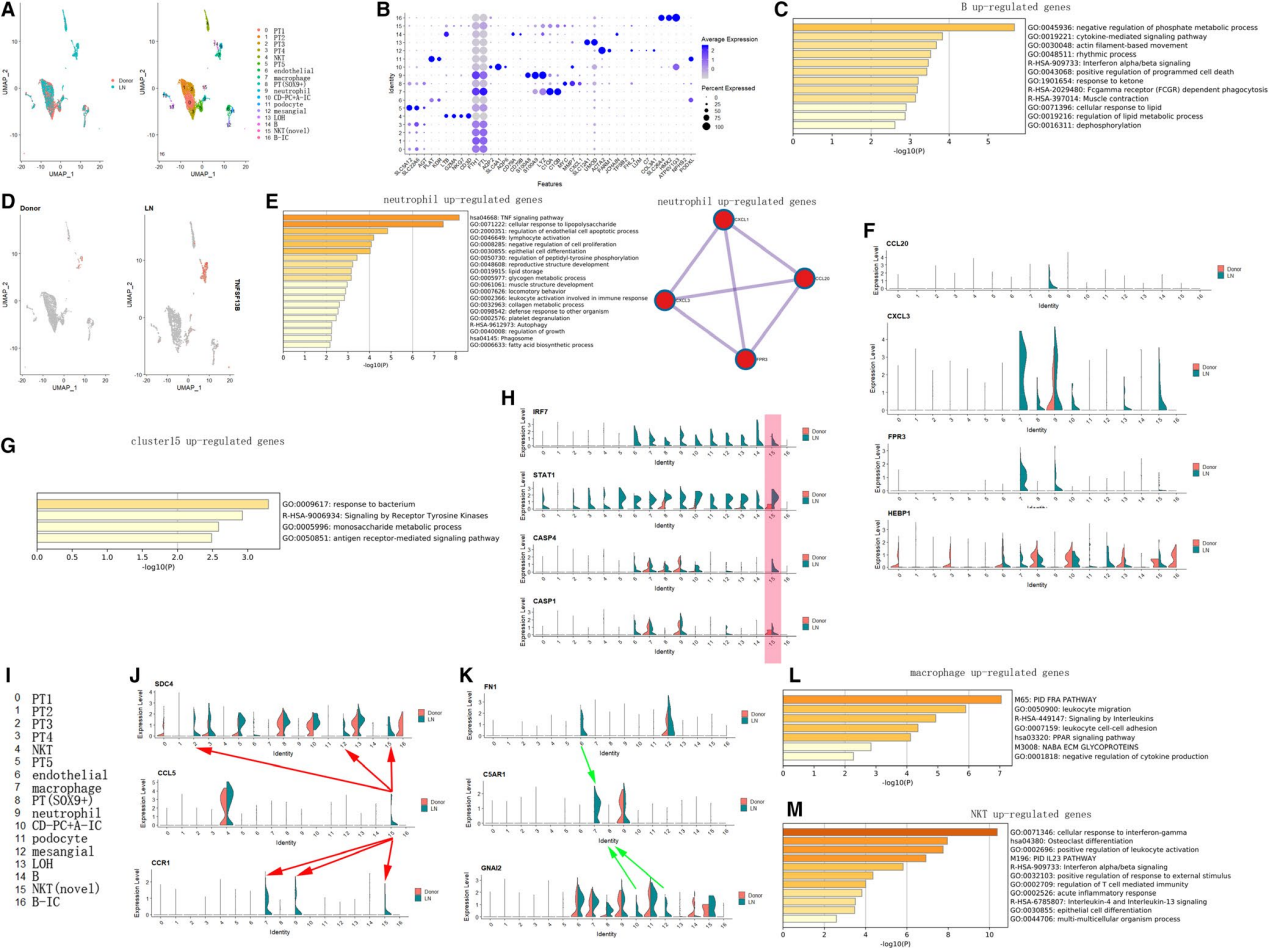
LN‐associated genes at the cellular level were shown. (A) One LN and one healthy donor were merged. The UMAP showed the cell clusters among different samples. (B) The marker genes of each cell clusters. Whereas in LN sample, interferon signalling was activated in B(C) and NKT(M).^46^ (D)*TNFSF13B* was gathered in monocyte of LN. The enrichment analysis of up‐regulated genes (p value < 0.05) of LN compared with donor control within neutrophil(E) was shown. The interaction of chemokines and the receptor, which was up‐regulated in neutrophil of LN compared with that of donor, was also showed. (F) The differential expression level of *CXCL3*, *CCL20*, *HEBP1* (a ligand of *FPR3*) and *FPR3* between LN and donor was shown by vlnplot. (G) The enrichment analysis of up‐regulated genes (p value < 0.05) of LN compared with that of donor control within cluster 15 was shown. (H) Pyroptosis associated genes were activated in cluster 15 of LN comparing with that of donor. (I) The name of each cell cluster. (J) (K) Vlnplot commonly showed the activated ligands and receptors interactions of LN compared with that of donor at cellular level. The enrichment analysis of up‐regulated genes (p value < 0.05) of LN compared with donor control within macrophage (L) and NKT(M) was shown

To investigate cellular‐specific LN‐associated genes, one LN and one healthy donor were merged. The UMAP showed the cell clusters among different samples (Figure 4A). The marker genes of each cell clusters were shown by dot plot (Figure 4B),whereas in LN sample, interferon signalling was activated in B (Figure 4C) and NKT (Figure 4M).^14^ *TNFSF13B* was gathered in the macrophage, neutrophil and B of LN( Figure 4D). Some chemokines such as *CXCL3* and *CCL20* can interact with *FPR3* to recruit more neutrophils to LN kidney (Figure 4E). Interestingly, a novel cell cluster having properties of NKT seems to undergo pyroptosis, as *IRF7/STAT1/CASP4* signalling was activated in this cell cluster (Figure 4H). The enrichment analysis of up‐regulated genes (p value < 0.05) of LN compared with donor control within neutrophil (Figure 4E) was shown. The differential expression level of *CXCL3*, *CCL20*, *HEBP1* (a ligand of *FPR3*) and *FPR3* between LN and donor was showed by vlnplot(Figure 4F). The enrichment analysis of up‐regulated genes (p value < 0.05) of LN compared with donor control within cluster 15 was shown (Figure 4G). Vlnplot commonly showed the activated ligands and receptors interactions of LN compared with the donor at cellular level (Figure 4J and 4K). The enrichment analysis of up‐regulated genes(p value < 0.05) of LN compared with donor control within macrophage (Figure 4L) and NKT (Figure 4M) was shown. These data declared that specific markers in LN were found at cellular level and the critical role of interferon alpha/beta signalling pathway was enriched in B and NKT of LN.

## 4 DISCUSSION

We have presented the first, to our knowledge, large‐scale scRNA‐seq study of the human glomerulonephritis, which has revealed previously unknown cellular diversity and subtype‐specific gene dysregulation in glomerulonephritis. This study wants to bring kidney biopsy interpretation into single‐cell transcriptomics of human glomerulonephritis.^15^

Firstly, as we know, PT cells were mainly divided into three subtypes: S1, S2 and S3. While in this data, *SLC5A2* (the marker of S1), *SLC22A6* (the marker of S2) and S3 marker gene *AGT* ^16^ did not show obvious differential expression across PT cell clusters(Extended figure 2). Decreased PT cell clusters and increased immune cell clusters in diseased samples comparing with two donors were found. This indicates that normal PT cell numbers were decreased in diseased patients. Indeed, PTs are susceptible to the derangements of some nephritis such as diabetic kidney disease^17^ and are vulnerable to various injuries. Additionally, PTs are the primary sensor and effector in the progression of chronic kidney disease (CKD).^18^

As there were more immune cells infiltrated in tissues from glomerulonephritis than the healthy donor, we analysed several disease associated cell clusters and defined some novel genes in the immune cells. Interestingly, one NKT like cell cluster high expressing *LTB* was found, which is consistent with the report from Humphreys lab, and *LTB* level was higher in lymphocyte of diabetes than that of control.^19^, ^20^, ^21^ As an inducer of the inflammatory response system, *LTB* was also involved in normal development of lymphoid tissue.^22^ From our data, high expressing *LTB* cell cluster was enriched in LN and one IgAN samples (Figure 1D), the up‐regulated genes in this cluster of IgAN comparing with that of donor was enriched in B cell activation signalling pathway, this suggests *LTB* high cell cluster may have a role in B cell activation to induce more immune response. In lgAN and CIN samples, the higher expression of TPSB2 and TPSAB1 in mast cells contributes to profibrogenesis ^23^, ^24^, ^25^; thus, there are more interstitial fibrosis in IgAN and CIN patients (Extended Figure 1 and Figure 1D). NKT was enriched in LN, and its high expressed genes were enriched in interferon alpha/beta signalling pathway^11^, ^26^ which was activated in LN from RNA‐seq data. Neutrophil was enriched in LN and CIN samples compared with other samples, suggesting innate immune response may involve in the process of LN and CIN, whereas more B and plasma infiltrate IgAN and CIN samples, suggesting antibody mediated immune response may involve in the process of IgAN and CIN.

Although, many GWAS studies have explored the correlation of gene SNP and glomerulonephritis, the cause and effect is still unclear. And we find GWAS genes focus on clusters of glomerulus and immune cells. From this, we can know glomerulonephritis was most relevant with the malfunction of glomerulus and immune cell clusters. Further study can focus on overactive immune responses and glomerulus function shortage both contribute to the disease occur and develop. As we know, there already exist some effective biologics targeting promising molecular, in order to explore the expression pattern of these molecular. From the Figure 1F, we can find that the major side effect of some biologics was not only because of deleting the normal function of target genes but also affecting target genes of various cell types as *CD52*, *CD74*, *MIF* and *TNFRSF12A* were displayed across various cell types. So biologics targeting these genes may have some side effects.

CD74 is a protein trafficking regulator and a cell membrane receptor for macrophage migration inhibitory factor (MIF).^27^ In our data set, four glomerulonephritis samples including two IgAN, one LN and one MN showed higher *CD74* mRNA level comparing with donor sample within podocyte, which indicates activation of *MIF/CD74* between immune cells and podocyte exists in glomerulonephritis sample, providing a potential marker for glomerulonephritis status.

Although FXYD proteins belong to a family of small‐membrane proteins, among them, FXYD5 (also called RIC) belongs to FXYD family, which is one of the subunits of Na‐K‐ATPase.^28^ It can reduce cell adhesion by down‐regulating E‐cadherin.^29^ In our data, we found *FXYD5* was enriched in podocyte of glomerulonephritis, which needs further study to declare the role of FXYD5 within podocyte in the progression of glomerulonephritis. As the nature of ß2‐microglobulin (*B2 M*) is pro‐inflammatory, its mRNA expression in cells of the urinary sediment is higher in DKD and FSGS in comparison with healthy patients, which may be reflecting to a tubulointerstitial injury promoted by albumin,^30^ and in our results, *B2 M* is high expressed in podocyte of glomerulonephritis rather than tubule, so the role of *B2 M* in podocyte needs to be further investigated.

In our results, chloride intracellular channel 1 (*CLIC1*) and *RPS26* were found to be up‐regulated in mesangial of IgAN with *JUNB* up‐regulated in podocyte of IgAN compared with those of donor. Ion channels form a large class of drug targets for human diseases. As either a soluble cytosolic or a membrane protein, oxidation, low pH and cholesterol‐rich lipid rafts can increase CLIC1 channel activity. CLIC1 is localized in intracellular organelles such as phagosomes in macrophage and dendritic cells, where it is thought to increase phagosome chloride concentration to counterbalance the charge difference induced by proton entry,^31^ so the increased *CLIC1* level in IgAN may add chloride concentration to phagosome, the cause of which need to be clarified further. RPS26 is a ribosomal subunit structural protein involved in the growth and development process.^32^ Little is known about the function of *RPS26* in kidney disease. We here only provide a clue of increased gene expression in mesangial of IgAN for further studies. However, why *CLIC1* and *RPS26* expression level increased in mesangial of IgAN needs further biological study to clarify. Upon receiving an external stimulus, the gene *JUN*, as an early responding transcription factor, is rapidly activated and dimerizes with either another JUN protein or with AP‐1 to form the FOS protein. As a gene structurally and functionally very similar to *JUN*, the gene *JUNB* along with JUN and FOS forms the upstream element of the TNF/TNFR1 pathway. TNF/TNFR1 pathway is also involved in RAAS activation, complement activation, coagulation cascades and inflammation, and one bioinformatics paper concludes that JUNB are maybe novel prognostic biomarker of IgAN,^33^ and similar to above result, *JUNB* was activated in podocyte of IgAN comparing with that of donor, leading to podocyte injuries.

Some MN high expressed genes such as *HLA‐DRB5*, *HLA‐DQA2*, *IFNG*, *CCL2* and *NR4A2*, which involve in highest enrichment pathway, were shown by vlnplot to display the cellular‐specific expression style. In cluster 12(macrophage), *HLA‐DRB5*, *HLA‐DQA2*, *CCL2* and *NR4A2* were hyper‐activated in MN comparing with donor. Besides in cluster 12, *HLA‐DRB5* was up‐regulated in cluster 4(endothelial), 13(endothelial), 14(neutrophil) and 17(mesangial) of MN compared with those of donor, whereas *IFNG* is specially higher expressed in cluster 16(NK) of MN. HLA class II antigens, which regulate the immune response, correlated with glomerulonephritis, including membranous nephropathy (MN) and IgA nephropathy. Furthermore, MN was significantly associated with HLA‐DRB5*0101 in Japanese patients.^34^ Our result showed *HLA‐DRB5* was up‐regulated in MN especially in macrophage, endothelial, neutrophil and mesangial, indicating that the immune response was activated in those cells of MN. CCL2, a pro‐inflammatory cytokine, participates in recruitment of monocytes and effector T cells to the sites of tissue injury,^35^ and in our results, the cellular origin of high expression of *CCL2* in MN was macrophage. Exogenous expression of *Nr4a2* in macrophages leads to its phenotype with induction of M2 marker genes. As we know, M2 like macrophage seems to promote the repair after injury, so the *NR4A2* increases its level in macrophage of MN, which is maybe the result of MN development.

While in LN sample, interferon signalling was activated in immune cells including B and NKT. Hyper‐activation of type I interferon (IFN‐I) signalling pathway is associated with the progression and prognosis of LN.^36^ Many molecules,^37^, ^38^, ^39^ such as JAK inhibitor (tofacitinib), monoclonal antibodies targeting IFN alpha (sifalimumab) and IFN‐I receptor (anifrolumab), by blocking IFN‐I signalling pathway have been developed to ameliorate the symptoms of SLE. As this data show, interferon signalling was activated in immune cells of LN comparing with that of the donor, so targeted therapy should be applied to over‐activation of interferon signalling within immune cells.

Interestingly, it was found that *TNFSF13B* (also called *BAFF*) was exclusively expressed in macrophage, neutrophil and B cells. Previous study found the levels of *BAFF* were significantly higher in active LN compared to inactive one,^40^ and from our data, the *BAFF* level within macrophage, neutrophil and B cells may be relatively higher in LN comparing with other kidney disease and donors. One clinical study comes to a conclusion that combined B cell targeted therapy with rituximab (RTX; anti‐CD20) and belimumab (BLM; anti‐BAFF) prevented full B cell repopulation including double negative B cells, with concomitant specific reduction of SLE‐relevant autoantibodies.^41^ So the high level of BAFF in macrophage, neutrophil and B cells may be a good biomarker for the activity of LN. Previous studies have shown lupus‐prone mouse macrophages were reported to display impaired lysosomal maturation that can lead to accumulation of nuclear antigens to the cell surface that can potentially activate autoreactive lymphocytes^42^; however, this study found there exist NKT like cells undergoing pyroptosis in LN kidney as *IRF7/STAT1/CASP4* expressed higher in the NKT like cell cluster of LN compared with that of donor control. One clinical study showed urine and serum levels of CCL5 were elevated in active LN as compared with disease remission,^43^ and our data showed *CCL5* expressed in NKT like cell cluster can interact with the receptor *SDC4* expressed in PT, mesangial and NKT like cell cluster, whereas *CCL5* interact with the receptor *CCR1*, which is expressed in macrophage and neutrophil.

A limitation of this study is the relatively small number of patients, and the sample size should be increased in future studies to better delineate the occurrence, development and outcome of kidney diseases. Our results lay the foundation for such efforts.

## 5 CONCLUSION

Single‐cell RNA‐seq is a powerful tool for mining kidney disease‐associated genes and the activated signalling pathways genes involved at the cellular level. By scRNA‐seq, we first defined the podocyte markers of glomerulonephritis and specific markers in IgA, MN and LN were found at cellular level. Furthermore, the critical role of interferon alpha/beta signalling pathway was enriched in B and NKT of LN was declared and such data sets of glomerulonephritis were provided in this paper to some extent.

## CONFLICT OF INTEREST

The authors declare that they have no competing interests.

## AUTHOR CONTRIBUTION

**zhejun chen:** Investigation (equal); Writing‐original draft (equal). **ting zhang:** Investigation (equal). **kaiqong mao:** Investigation (equal). **xinghua shao:** Investigation (supporting). **yao xu:** Investigation (supporting). **minyan zhu:** Investigation (supporting). **hang zhou:** Investigation (supporting). **qin wang:** Investigation (supporting). **yuanyuan xie:** Investigation (supporting). **zhenyuan li:** Investigation (supporting). **ming zhang:** Investigation (supporting). **xiaodong yuan:** Investigation (supporting). **liang ying:** Investigation (supporting). **jiajia hu:** Supervision (equal). **shan mou:** Supervision (lead).

## ETHICS APPROVAL AND CONSENT TO PARTICIPATE

The patient described in this study consented under ethics committee review of Renji hospital affiliated to Shanghai Jiao Tong University School of Medicine.

## Supporting information

The split view of cell clusters at the level of different samples. This plot roughly shows the proportion of 20 cell clusters in the respective kidney sample.

The expression of known PT markers across different PT types.^1‐3^ This plot shows the five PT types whose properties were defined by the expression level of the known PT markers.

The mRNA expression level of CD74，B2M and FXYD5 between control and IgAN or LN. A higher mRNA level of CD74, B2M and FXYD5 in IgAN or LN kidney sample comparing with control living donor was found in the Renal Gene expression database (http://rged.wall-eva.net/).

## Data Availability

The data sets generated during the current study are available for uploading if required.
