# Supplementary material for: A single‐cell survey of the human glomerulonephritis
Source: J Cell Mol Med. 2021 Mar 22;25(10):4684–95. doi: 10.1111/jcmm.16407 (PMC8107090; doi:10.1111/jcmm.16407)
Supplement: Supplementary file 1 — The split view of cell clusters at the level of different samples. This plot roughly shows the proportion of 20 cell clusters in the respective kidney sample. [file JCMM-25-4684-s003.pdf]

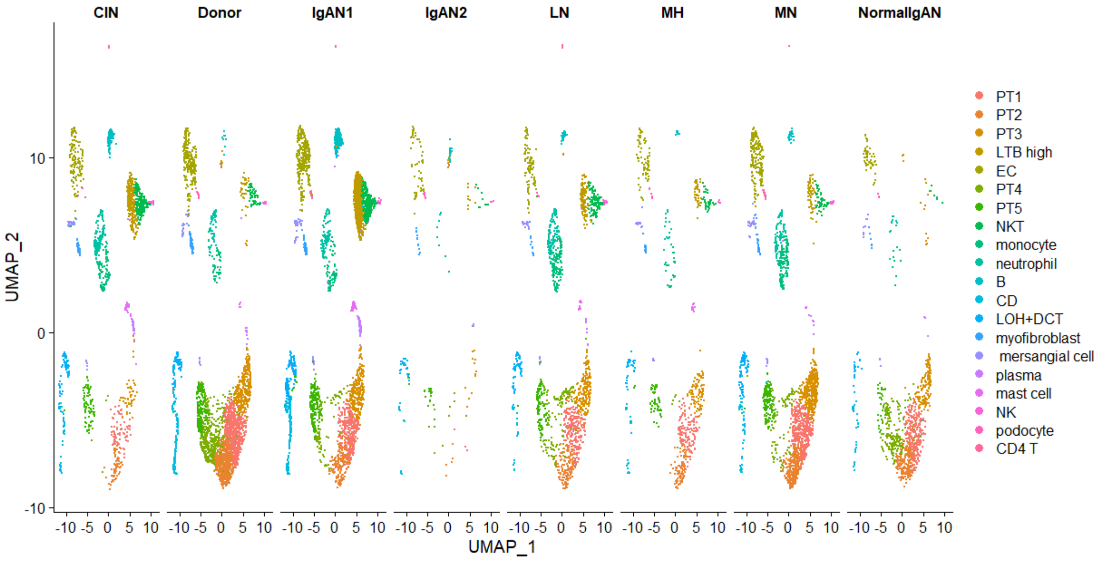

Extended figure1 The split view of cell clusters at the level of different samples. This plot roughly shows the proportion of 20 cell clusters in the respective kidney sample.
