# Supplementary material for: A single‐cell survey of the human glomerulonephritis
Source: J Cell Mol Med. 2021 Mar 22;25(10):4684–95. doi: 10.1111/jcmm.16407 (PMC8107090; doi:10.1111/jcmm.16407)
Supplement: Supplementary file 2 — The expression of known PT markers across different PT types.1‐3 This plot shows the five PT types whose properties were defined by the expression level of the known PT markers. [file JCMM-25-4684-s002.pdf]

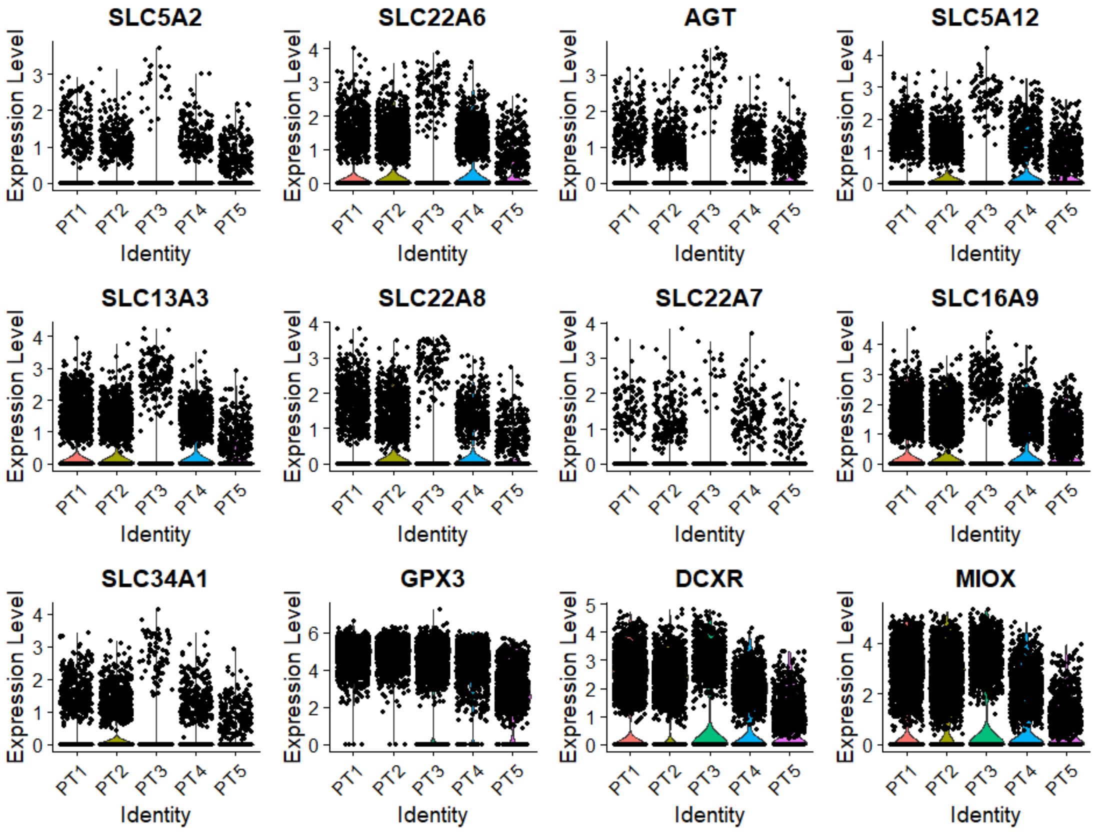

Extended figure2 The expression of known PT markers across different PT types (1-3).This plot shows the five PT types whose properties was defined by the expression level of the known PT markers.

1. Young MD, Mitchell TJ, Vieira Braga FA, Tran MGB, Stewart BJ, Ferdinand JR, et al. Single-cell transcriptomes from human kidneys reveal the cellular identity of renal tumors. Science (New York, NY). 2018;361(6402):594-9.
2. Stewart BJ, Ferdinand JR, Young MD, Mitchell TJ, Loudon KW, Riding AM, et al. Spatiotemporal immune zonation of the human kidney. Science (New York, NY). 2019;365(6460):1461-6.
3. Liao J, Yu Z, Chen Y, Bao M, Zou C, Zhang H, et al. Single-cell RNA sequencing of human kidney. Scientific data. 2020;7(1):4.
