# Supplementary figures and images for: A single‐cell survey of the human glomerulonephritis
Source: J Cell Mol Med. 2021 Mar 22;25(10):4684–95. doi: 10.1111/jcmm.16407 (PMC8107090; doi:10.1111/jcmm.16407)

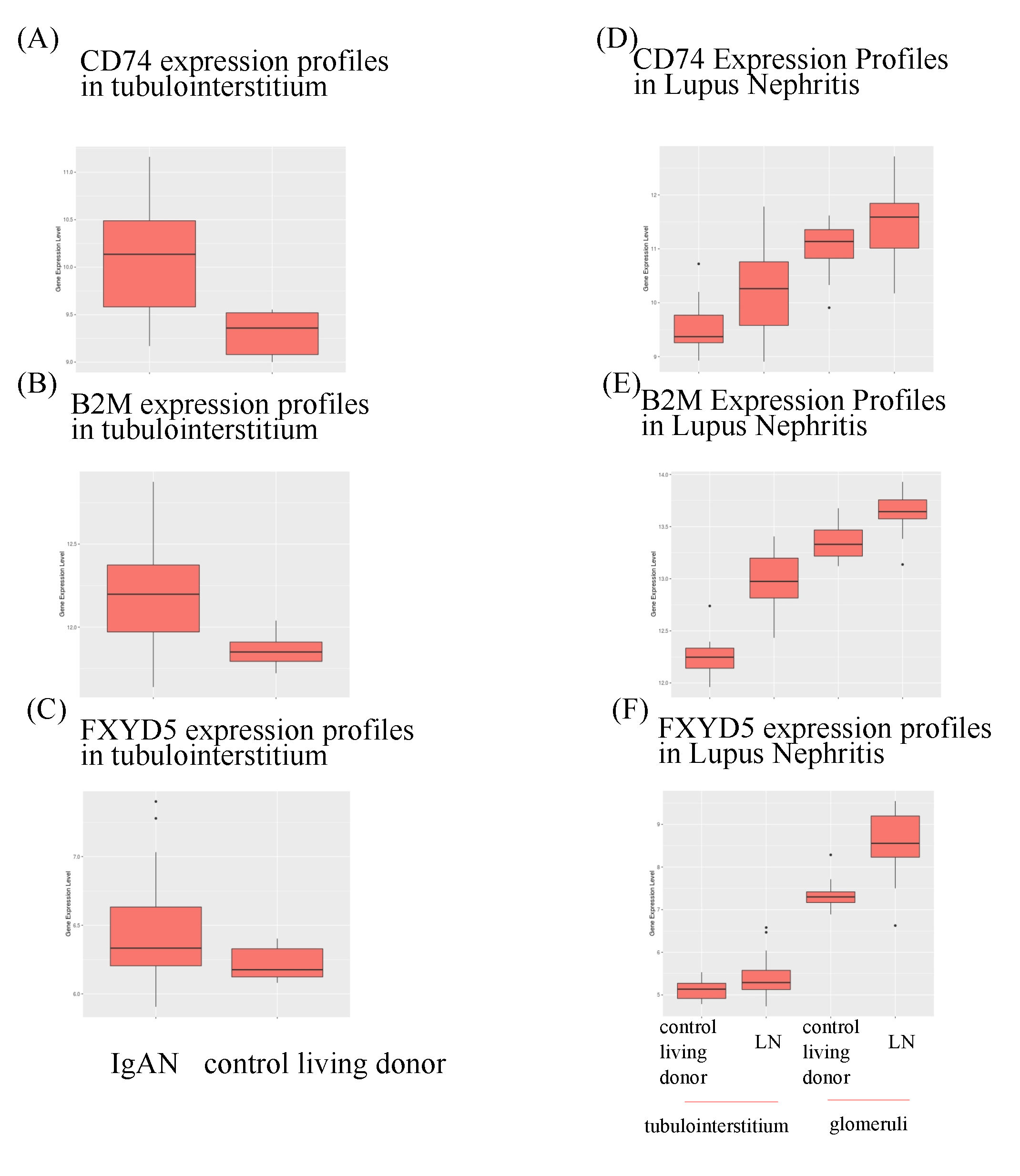

Supplement: Supplementary file 3 — The mRNA expression level of CD74，B2M and FXYD5 between control and IgAN or LN. A higher mRNA level of CD74, B2M and FXYD5 in IgAN or LN kidney sample comparing with control living donor was found in the Renal Gene expression database (http://rged.wall-eva.net/). [file JCMM-25-4684-s001.tif]
